# *Tos17* rice element: incomplete but effective

**DOI:** 10.1186/1759-8753-5-10

**Published:** 2014-04-01

**Authors:** Francois Sabot

**Affiliations:** 1 UMR DIADE IRD/UM2, 911 Avenue Agropolis BP64503, F-34394 Montpellier Cedex 5, France

## Abstract

**Background:**

*Tos17* was the first LTR retrotransposon (*Copia*) described as active in cultivated rice, and is present in two copies in the genome of the sequenced Nipponbare variety. Only the chromosome 7 copy is active and able to retrotranspose, at least during *in vitro* culture, and this ability was widely used in insertional mutagenesis assays.

**Results:**

Here the structure of the active *Tos17* was thoroughly annotated using a set of bioinformatic analyses.

**Conclusions:**

Unexpectedly, *Tos17* appears to be a non-autonomous LTR retrotransposon, lacking the *gag* sequence and thus unable to transpose by itself.

## Background

The long terminal repeats (LTR) retrotransposon life cycle involves a cytosolic reverse-transcription step within a multiproteic core called virus-like particle (VLP), formed by the polymerization of the Group-specific antigen (GAG) proteins, normally encoded in the element itself; for a recent review, see [1]. This GAG protein classically harbors three domains, from external to internal:

1) the matrix domain (MA), for membrane targeting and capsid assembly;

2) the capsid hydrophobic region (CA) and the most conserved part of GAG, in charge of polymerization, and the

3) nucleocapsid (NC), targeting the specific mRNA through the PSI region [1].

In addition, a CCHC zinc-finger motif is located at the C-terminus of the protein, single or twice repeated (or even thrice), and is in charge of the protein-nucleic acid interactions [1]. This protein is theoretically specific of its own RNA, and is an essential and mandatory component of the retrotransposition of LTR retrotransposons. A second open reading frame (ORF), *pol*, encodes the reverse transcriptase-RNaseH (RT-RNaseH), which drives the synthesis of a double-stranded cDNA from two RNA matrices and the integrase (INT) which allows the insertion of the new cDNA copy. However, in some cases, some non-autonomous elements have been shown capable of hijacking the GAG from other elements [2].

In cultivated Asian rice (*Oryza sativa* L.), LTR retrotransposons compose at least 20% of the genome (MSUv7.0 reference genome [3], http://rice.plantbiology.msu.edu/index.shtml). The *Copia Tos17* element (for Transposon of *Oryza sativa* 17) was the first identified as active [4] and able to transpose in this genome. Moreover, *Tos17* seems to be the most transpositionally competent one in regenerated plants [5].

**Figure 1:**
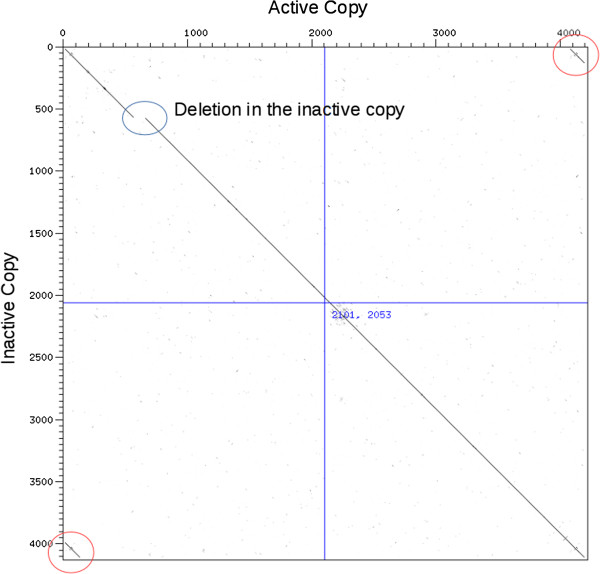
**Dotter alignment of the two *Tos17 *genomic copies.** Horizontally, the copy from chromosome 7 (active copy); vertically, the copy from chromosome 10 (inactive copy). The difference in transcriptional/transpositional activity is due to small mismatches, forming stop-codons and frameshifting, and to a larger deletion in the inactive copy (blue circle). The long terminal repeats (LTR) repeated sequences are circled in red.

Two almost identical genomic copies of *Tos17* reside in the reference genome (on chromosomes 7 and 10; Figure 1). Only the chromosome-7 copy is transpositionally active (during *in vitro* culture at least), whereas the other, located on chromosome 10, is inactive, heavily methylated and contains several stop codons and indels in its predicted coding region [6]. This last copy can, however, be reactivated (transcriptionally) in methylation-defective mutants [6]. In the whole *Oryza* genus, the copy number as well as the location of active copies (if there are any) may differ [7].

The *Tos17* activation during *in vitro* culture was widely used in mutagenesis assays, which allowed reverse genetics analyses through the generation of insertional mutants without transformation [8-10]. In the present study, a detailed functional analysis of *Tos17* was performed, showing that both genomic *Tos17* copies lack a *gag* ORF, making *Tos17* a non-autonomous element requiring an active one in order to ensure its transposition.

## Results and discussion

**Figure 2:**
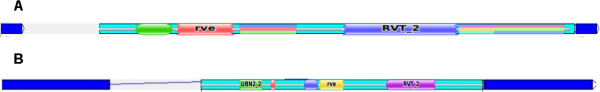
**Annotation of *Tos17 *and *RIRE1*.** Long terminal repeats (LTR) are symbolized in dark blue and the open reading frame (ORF) in light blue. **(A)***Tos17* annotation. The *gag_pre-integrase* (green), *rve* (red) and *RVT_2* (blue) motif positions are reported on the ORF. **(B)***RIRE1* annotation. The *UBN2_2* (green), Zf-CCHC (red), *gag_pre-integrase* (blue), *rve* (yellow) and *RVT_2* (purple) motif positions are reported on the ORF.

The two *Tos17* genomic copies were extracted from their respective location in the rice MSUv7.0 genome, and manually annotated using a series of basic local alignment search tool (BLAST), ProSite and Protein families (Pfam) analyses. A predicted long ORF (from position 659 to 3835, Figure 2A; annotated as the *gag-pol* ORF [4]) of 1,058 residues can be detected on the active copy (chromosome 7), whereas no apparent ORFs (that is, more than 100 residues starting with Met) exist on the inactive copy. On this long ORF, INT (*gag_pre*-*integrase* and *rve*) and RT (*RVT_2*) *Pfam*-A motifs can be easily identified (see Table 1), which suggests that this ORF is the polyprotein (POL) one. However, none of the truly GAG-related motifs, such as CCHC zinc-finger (18 residues) or the *UBN2* group (100 to 150 residues), could be identified, and the first confidently identified motif related to the INT (and thus to the pol ORF) in the *Pfam* database starts at residue 79 (Figure 2A; *gag_pre*-*integrase* motif) of this ORF (base 757 of the internal sequence).

**Table 1:** **Tos17 Open reading frame (ORF)2 *Pfam *** **motifs**

**Motif**	**Start**	**Stop**	**e-value**
gag_pre-integrase PF13976	79	153	6.50e-016
rve PF00665	164	284	3.60e-026
RVT_2 PF07727	519	762	1.10e-095

The *Pfam* analysis was performed on the largest *Tos17* ORF.

**Figure 3:**
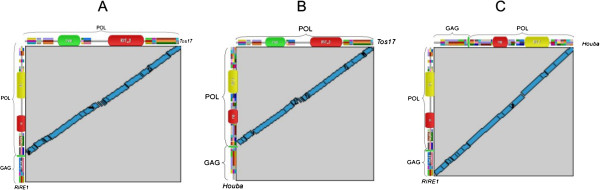
**Structural comparisons.** Comparison between the protein structures of **(A)***RIRE1* and *Tos17*, **(B)***Houba* and *Tos17*, and **(C)***RIRE1* and *Houba*. In **A** and **B**, no GAG region is found in *Tos17*, homologies being limited to the polyprotein (POL) part.

This ORF was then compared to ORFs from those of the active *Copia* elements, *RIRE1* from *Oryza. australiensis*[11,12] [BAA22288; EMBL/GB] (Figure 2B), and *Houba* from *O. sativa* (known to be one of the most recently retrotransposed *Copia* in rice; [13]). As shown on Figure 3, the ORFs aligned on the whole POL part the elements that are compared two by two; the *Tos17* ORF, however, lacked the GAG region, while the ORFs from *RIRE1* and *Houba* are also aligned on the GAG part. No GAG-related region can be detected on the whole *Tos17* genomic sequences in *BLASTx* against *nr* and protein databases (data not shown). Various *tBLASTn* (protein query versus nucleic database) analyses against the rice EST databases from NCBI were performed, and no ESTs resembling a larger ORF than the ones known were detected. Finally, no other *Tos17 gag*-like sequence can be amplified in PCR on the NipponBare genomic DNA (data not shown).

RT-sequence phylogenetic analysis showed that only *RN304* and *Lullaby* are closely related to *Tos17*[14]. Interestingly, *RN304*, the closest element to *Tos17*, is itself also a non-autonomous element also lacking the *gag* sequence, similar to *Tos17*, but no information about its transpositional activity is available. The closest complete element to *Tos17* (that is, one that harbors a complete g*ag-pol* ORF) is the *Lullaby* element, recently shown as transitionally active in only some of the regenerated lines in which its expression was detected [14]. *Lullaby* is a 5′142-long element, and has two copies in the Nipponbare genome, on chromosomes 6 and 9, with only the chromosome-6 copy active [14]. The DNA similarity between *Tos17* and *Lullaby* is 57% at the DNA level (whole element sequence), and 64% at the protein level (*gag-pol* region sequence). At the DNA level, the similarity is limited to the internal sequence, whereas at the protein level the two POL sequences aligned well. Moreover, the primer binding site (PBS) region, located immediately after the 5′ LTR, and involved in RNA-GAG recognition [1], is almost identical between the two elements (5′-TGGTATCAGAGC(a/t)A(t/-)GGT-3′), starting at positions 126 and 139 for *Lullaby* and *Tos17* respectively. However, no common INT signal (at the 3′-end of the 3′ LTR [1]) is shared between *Lullaby* and *Tos17*, highlighting the use of *Lullaby* GAG by *Tos17* only.

*Tos17*, the most active LTR retrotransposon in cultivated rice, and the most commonly used element as an insertional mutation tool [8], is thus a non-autonomous element, because no *gag* sequence exists in the *Oryza sativa* genome, even if *Tos17* is able to retrotranspose in this species. The simplest explanation is that *Tos17* is coupled with an active LTR retrotransposon for its mobility, and that the former is able to use the *gag* (and VLP) from the latter. Such hitchhiking implies a structural (same GAG-recognition signals) as well as translational (same time of expression) relationship between *Tos17* and its autonomous partner. This association is probably a long-term association, as the structural annotation of the *Tos17* elements (Figure 2A) reveals a complete removal of the *gag* region, without any identifiable remnants, but without damaging any other structural features of the element (LTR, PBS or polypurine tract (PPT)). Indeed, such clean elimination might have occurred during *Tos17* evolution, with only elements within this correct deletion selected (able to be correctly expressed and mobilized by its partner), as no other *Tos17*-like element with *gag* remnants has been detected.

The use of *Tos17* as an insertional tool for reverse genetics is not affected by this non-autonomous state, as long as requested functional and complementation analyses are performed to validate or invalidate the insertion as the real cause of the observed phenotype. The fact that *Tos17* is not able to retrotranspose by itself may help to explain the high rate (almost 90%) of morpho-physiological variations untagged by *Tos17* (or the transferred T-DNA) observed among regenerated lines ([9]; M Lorieux, unpublished data; B Hsingh, personal communication), which is probably also due to transposition of other elements, as shown previously [5].

## Conclusion

Analyses, such as the one described here, highlight the need for a better knowledge of transposable elements (TEs), in order to ensure a better understanding of their effects upon the host genome. In particular, it may be of interest to further study the details of the relationships between the non-autonomous elements and their autonomous counterparts, because existing data suggest that the former are more active than the latter, as shown for *BARE2* and *Tos17*.

## Methods

The nucleotidic sequences from genomic copies of each element were launched in Artemis [15], and the ORFs longer than 100 residues were automatically extracted from the element sequences. The ORFs were then scanned online using a combination of Pfam*,* ProSite and BLASTp analyses [16] with standard parameters. The results were then reported on Artemis, in order to manually reconstruct the complete structure of each element. The LTRs were identified using Dotter [17], and the PBS and PPT were manually determined. The comparison between putative GAG-POL sequences was performed using the Align2Sequence graphical tool from the NCBI, through a BLASTp analysis, for a better presentation. The identity/similarity levels were calculated using the Stretcher program from the EMBOSS suite.

## Abbreviations

BLAST: basic local alignment search tool; GAG: group-specific antigene; INT: integrase; LTR: long terminal repeats; ORF: open reading frame; PBS: primer binding site; POL: Polyprotein; PPT: Polypurine tract; TE: transposable element; Tos: transposon of *Oryza sativa*; VLP: virus-like particle.

## Competing interests

The author declares having no competing interests.
